# Impact of Emerging Technologies on Virgin Olive Oil Processing, Consumer Acceptance, and the Valorization of Olive Mill Wastes

**DOI:** 10.3390/antiox10030417

**Published:** 2021-03-09

**Authors:** Maria Pérez, Anallely López-Yerena, Julián Lozano-Castellón, Alexandra Olmo-Cunillera, Rosa M. Lamuela-Raventós, Olga Martin-Belloso, Anna Vallverdú-Queralt

**Affiliations:** 1 Department of Nutrition, Food Science and Gastronomy XaRTA, Institute of Nutrition and Food Safety (INSA-UB), Faculty of Pharmacy and Food Sciences, University of Barcelona, 08028 Barcelona, Spain; mariaperez@ub.edu (M.P.); naye.yerena@gmail.com (A.L.-Y.); julian.lozano@ub.edu (J.L.-C.); alexandra.olmo@ub.edu (A.O.-C.); lamuela@ub.edu (R.M.L.-R.); 2 Laboratory of Organic Chemistry, Faculty of Pharmacy and Food Sciences, University of Barcelona, 08028 Barcelona, Spain; 3 CIBER Physiopathology of Obesity and Nutrition (CIBEROBN), Institute of Health Carlos III, 28029 Madrid, Spain; 4 Department of Food Technology, Agrotecnio Center, University of Lleida, Av. Alcalde Rovira Roure, 191, 25198 Lleida, Spain

**Keywords:** oil yield, phenols, volatile compounds, oxidative stability, circular economy

## Abstract

There is a growing consumer preference for high quality extra virgin olive oil (EVOO) with health-promoting and sensory properties that are associated with a higher content of phenolic and volatile compounds. To meet this demand, several novel and emerging technologies are being under study to be applied in EVOO production. This review provides an update of the effect of emerging technologies (pulsed electric fields, high pressure, ultrasound, and microwave treatment), compared to traditional EVOO extraction, on yield, quality, and/or content of some minor compounds and bioactive components, including phenolic compounds, tocopherols, chlorophyll, and carotenoids. In addition, the consumer acceptability of EVOO is discussed. Finally, the application of these emerging technologies in the valorization of olive mill wastes, whose generation is of concern due to its environmental impact, is also addressed.

## 1. Introduction

Extra virgin olive oil (EVOO), one of the key foods of the Mediterranean diet, is distinguished by its high content of nutritional and antioxidant compounds compared to other vegetable oils. It is composed mainly of triglycerides and more than 230 minor chemical compounds, although the composition varies depending on the variety, agronomic conditions, production processes, and various other factors [1,2,3]. The main minor compounds are aliphatic and triterpene alcohols, sterols, hydrocarbons, and antioxidants such as carotenoids and polyphenols, which are responsible for the organoleptic properties, stability, and nutritional value of EVOO [4,5]. 

There is a growing consumer demand for high quality EVOO, which is characterized by a high content of phenolic and volatile compounds with health-promoting and sensory properties. Critical parameters to obtain optimum quality EVOO with high antioxidant potential are the temperature and duration of the malaxation process. 

Considerable efforts have been dedicated to finding alternative processes that can preserve the quality attributes of foods, while being environmentally friendly and low in cost. As a result, several novel and emerging technologies have been developed and applied to satisfy the growing consumer demand for more natural products with fewer additives and preservatives that also offer convenience, freshness, and safety [6,7]. Modern food processing is based on advancements of traditional techniques (e.g., vacuum cooking, assisted thermal processing), as well as on the integration of novel procedures, mainly pulsed electric fields (PEF), high pressure processing (HPP), ultrasound (US), high-power ultrasound (HPU), and microwave (MW) treatments. These methods have been studied for their capability to enhance food products attributes, such as color; texture and flavor [8,9,10]; the contents of phenolic compounds, carotenoids, and vitamins; and also the availability of bioactive compounds [11,12,13]. In the production of EVOO, these techniques are based on rupturing the cell walls and membranes of the olive fruit and promoting pore formation and membrane permeability, which leads to water influx, swelling, and deflation. As a result, oil extraction during malaxation is improved and higher yields are obtained [13]. However, the application of these new technologies in EVOO production is still in its early days, and only a few preliminary studies have focused on their effects on oil yield and quality [14].

Regardless of the advantages of emerging food technologies, the market success of the product is highly dependent on acceptance by the consumer, who may have concerns about effects on health or the environment [15]. The food industry needs to challenge the common perception of new technologies as disruptive, expensive, and risky, and persuade the consumer of their benefits, which include competitive and low-margin food production. A primary factor in the consumer choice of food products is the perception of health benefits [16,17]. There is also a greater readiness to pay extra for new products if they are believed to have more quality and convenience [18]. As well as attributes of the product itself, production characteristics, such as origin, animal welfare, and production technology, influence consumer behavior.

The main initial focus in the development of emerging food technologies was meeting consumer demand for high quality, safe, nutritious, and minimally processed foods [19]. However, another concept has been gaining importance, the sustainability. Olive oil production generates huge quantities of waste products, known as olive mill wastes (OMW), which are phytotoxic and a major environmental concern. Although they have a negative impact on the environment, OMW have great potential as a source of beneficial compounds, such as phenolics, prompting many studies to investigate their recovery and valorization [20]. Nevertheless, the quantities of OMW generated are so high that their reduction remains a priority. A pertinent question is to what extent could the emerging technologies be more environmentally sustainable than conventional processes when applied in olive oil production. Broadly speaking, studies have shown that the application of those techniques can result in the reduction of energy and water consumption, and therefore reduce the carbon and water footprint of food processing [21]. 

The aim of this review is to provide an overview of the emerging technologies being applied to EVOO production and the results achieved so far. The sustainability of these techniques and the concerns they generate among consumers are also discussed.

## 2. Influence of Emerging Technologies on EVOO Production (Yield and Quality)

Inside the cells of olive fruits, the oil is partially located in the vacuole in a free form (approximately 76%), and the rest is found inside the cytoplasm, where it is dispersed as small droplets attached to colloids [22]. The conventional procedure for EVOO extraction includes a malaxation process, whose application increases yield compared to non-malaxated olives by approximately 5%, a significant improvement for the olive oil industries [23]. However, the temperature and duration of malaxation can compromise the quality of olive oils [23]. In the last decade, innovative mild techniques have been proposed to enhance EVOO production without a negative impact on the quality parameters. In Table 1, the effect of emerging technologies on yield, quality parameters, and bioactive compounds of EVOO is summarized.

### 2.1. Pulsed Electric Fields

Potential benefits of PEF have been demonstrated in recent research. Compared to thermal processing, PEF treatments are energy- and time-saving [22]. PEF treatments can be applied at high or moderate field strength. On the one hand, high-intensity PEF are an alternative to conventional food preservation techniques. The ability of high intensity PEF to obtain shelf-stable liquid foods with high nutritional value has been demonstrated [51]. On the other hand, moderate-intensity PEF permeabilize tissue structures, thus improving intracellular metabolite extraction [52] and enhancing drying efficiency [53]. Therefore, PEF-processed products could contribute to increasing the daily intake of health-promoting compounds [54]. 

**Figure 1:**
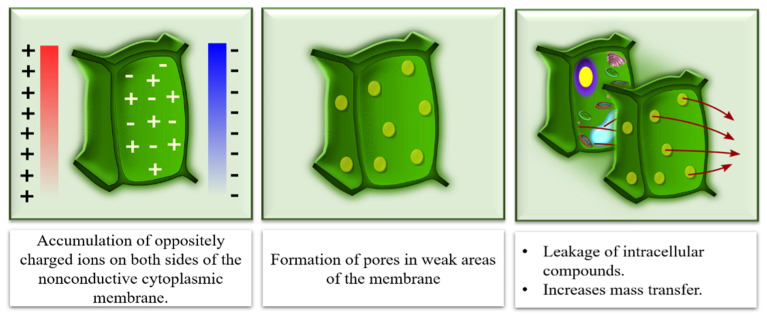
Effect of pulsed electric fields (PEF) treatment on cell membrane.

Electroporation, induced by the PEF treatment, exposes the cell membrane to an electric field, resulting in an increase in the transmembrane potential (accumulation of oppositely charged ions on both sides of the nonconductive cytoplasmic membrane) and the formation of pores in weak areas of the membrane [25,55]. The electroporation leads to leakage of intracellular compounds and increases mass transfer, as shown in Figure 1.

PEF applications have the potential to increase EVOO phytonutrient content and health-giving properties. Table 1 summarizes the parameters employed in PEF treatment during EVOO extraction. In a study on three different varieties of olive fruits (Tsounati, Amfissis and Manaki), different PEF intensities (1.6–70.0 kJ/kg) were applied before malaxation (30 min at 30 °C) [23]. Together with achieving an extraction yield of up to 18%, the treatment increased the total phenolic content and oxidative stability of the olive oil. In a comparative study on oil yields, four processes were applied to fresh blue olives: mild PEF (0.7 kV/cm), severe PEF (1.3 kV/cm), freezing-thawing, and thermal treatment at 50 °C for 30 min [27]. Freezing-thawing resulted in the highest oil yield (7.9%), but this treatment requires much more energy input than PEF. Regarding PEF, the yield was dependent on the field strength, being higher for the severe treatment (7.4%).

The effect of PEF of different intensities (0–2 kV cm^−1^) on Arbequina olive paste was studied along with a range of malaxation times (0, 15, and 30 min) and temperatures (15 and 26 °C) [22]. The extraction yield obtained without malaxation was improved by 54% after the application of the maximum PEF intensity (2 kV cm^−1^); when applied with malaxation at 15 °C, the improvement was 14.1%, whereas no effect was observed with malaxation at 26 °C. Therefore, the application of a PEF treatment allowed the malaxation temperature to be reduced from 26 to 15 °C, avoiding negative effects on extraction yield. In another study, the application of a PEF treatment of 16 kV of pulse voltage after olive crushing and before the malaxation step resulted in an increase in extractability of 3.71% and in yield of 0.38% [26].

The cell disintegration caused by PEF application to the olive paste allows malaxation to be carried out at a lower temperature, resulting in a better oil quality [56]. Accordingly, in addition to improving the oil extraction yield, a PEF treatment (2 kV cm^−1^ and frequency of 25 Hz) applied to Arroniz olive paste enhanced the EVOO quality in terms of polyphenol, phytosterol, and tocopherol contents [24]. A positive effect on extractability was also observed when olive paste from the Nocellara del Belice cultivar was treated with PEF (2 kV cm^−1^, 7.83 kJ/kg), leading to a 40.5% reduction in pomace oil loss without affecting the oil quality and causing a slight increase in the amount of oleacein and oleocanthal [25]. From the health point of view, high contents of secoiridoids are of interest due to their anti-inflammatory activity, which can be significant in many pathologies. Moreover, both oleacein and oleocanthal are responsible for the bitter and pungent taste of EVOO, respectively [5,57].

The application of PEF treatments in oil production from three Italian olive cultivars (Carolea, Coratina, and Ottobratica) by Veneziani et al. [13] resulted in improvements in yield (2.3% to 6%) and hydrophilic phenol concentration (3.2% to 14.3%). Importantly, the legal quality parameters or oxidative stability of the oil were not affected by the changes in the olive tissue structure induced by PEF. Likewise, the concentrations of α-tocopherol and the main classes of volatile compounds responsible for EVOO flavor were not significantly modified. The PEF technique was therefore able to improve oil extractability and antioxidant contents without negatively affecting the main qualitative and organoleptic characteristics of the final product. However, the performance of the PEF system may vary according to the particular geographical, morphological, and agronomical traits of the cultivar. More studies are required to assess the PEF effects, varying the machines and process parameters of the extraction plant [25].

### 2.2. High Pressure Processing 

**Figure 2:**
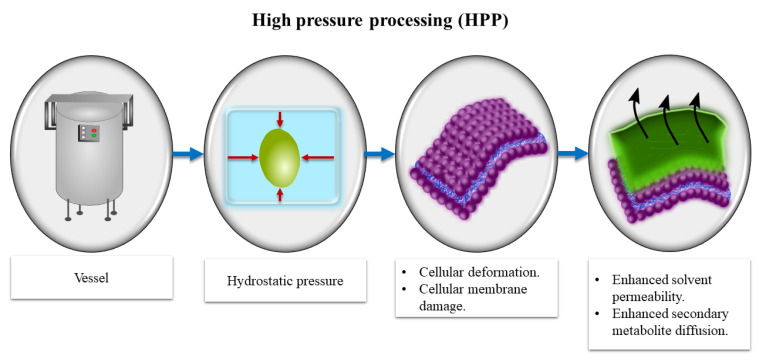
Cellular membrane deformation and damage caused by HPP.

The application of HPP can cause structural changes in foods, including cellular deformation and membrane damage [58], which may enhance solvent permeability in cells and secondary metabolite diffusion [59], as shown in Figure 2. HPP treatments stimulate mass transfer across the membrane due to differential pressure between the cell interior and exterior, which is followed by a quick re-establishment of an equilibrated concentration. There are few references of HPP technology being applied to increase the yields of EVOO. Andreou et al. [23] studied the effect of HPP (200 and 600 MPa, 25 °C for 1 and 5 min) used before malaxation (30 min at 30 °C) on three different varieties of olive fruits (Tsounati, Amfissis, and Manaki) and found an increase in extraction yield of up to 16%. Shelf-life tests indicate that the quality of oil from non-thermally pre-treated olives varies according to the conditions used, but oil produced from HPP-treated olives had a higher oxidative stability compared to control samples [23]. Therefore, HPP could potentially be applied to produce superior quality EVOO with increased yields. The combined application of filtration and high hydrostatic pressure on veiled EVOO has been studied. The resulting oil was not very susceptible to enzymatic and non-enzymatic phenomena, as it had no microbial contamination, a low water content, and low water activity, the opposite of when only a high hydrostatic pressure was applied [28]. 

### 2.3. Ultrasound Technology 

**Figure 3:**
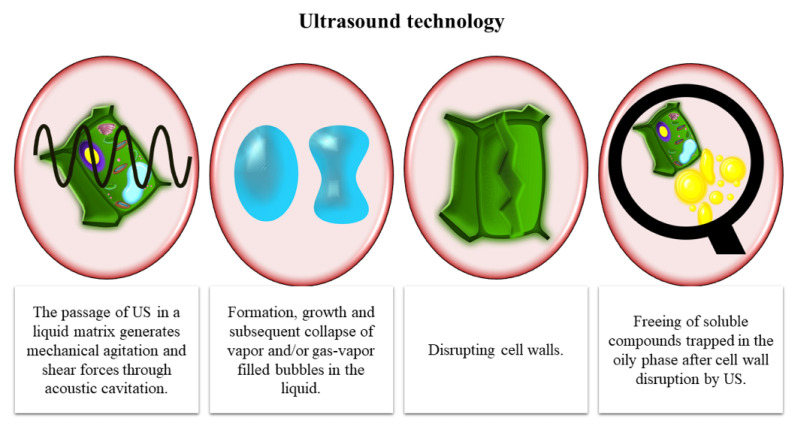
Freeing of soluble compounds trapped in the oily phase after cell wall disruption by ultrasound. US, ultrasound.

US consists of mechanical sound waves that arise from molecular oscillations in a propagation medium. Its potential in food processing has recently been harnessed in the development of several effective and reliable applications [60]. The passage of US in a liquid matrix generates mechanical agitation and shear forces through acoustic cavitation and results in an increase in mass transfer and the breakdown of cell walls [61] (Figure 3). When applied to olive paste before malaxation, US increased the efficiency of oil extraction by promoting the release of oil and minor compounds in the uncrushed olive tissue, thus reducing malaxation time [30] and production costs. However, its effectiveness could be limited, as the olive paste attenuates the transmission of the sound waves [29]. A recent study comparing US and PEF in terms of yield and extractability of olive oil found that the two technologies gave similar results, increasing both parameters in comparison with the untreated samples [26].

Clodoveo et al. [38] compared the effects of US on uncrushed olives submerged in a water bath and on olive paste. In both cases, the treatment reduced malaxation time and improved the quantity of minor compounds in the EVOO, although these effects were greater when treating whole olives. The effect of US applied to olive paste before malaxation was also studied [31]. For this purpose, two different Southern Italian olive varieties (Coratina and Peranzana) and a range of US treatment times (0, 2, 4, 6, 8, 10 min) were investigated. The resulting oil was assessed for sensory and other properties, including free acidity, the peroxide value, specific extinction coefficients K_232_ and K_270_, tocopherols, total carotenoids, chlorophyll, and total polyphenols. The longest US treatments (8 and 10 min) reduced the malaxation time from 60 to 40 min. Overall, the US technique improved the antioxidant content in both oil varieties, except for polyphenols. However, in a subsequent study by the same laboratory, a significant increase in polyphenols was observed in the sonicated oils, which was attributed to the effect of US on polyphenol oxidase activity [30]. In a recent research on the effects of US on the phenolic content of oil, the concentration of secoiridoids increased by 60% when using depitted olives, and this positive effect was enhanced when applying longer US treatment [32].

In a pilot-scale study, US treatments were applied to olive paste to determine if this emerging technology could enhance extraction yields, thereby achieving a more environmentally sustainable oil production [33]. A significant reduction in malaxation time was achieved, and when the extraction was carried out from the paste without malaxation the yield was higher compared to the control. Quality parameters (acidity, peroxide value, and K_232_ and K_270_) were not affected, although the EVOO produced from the treated olive paste was more pigmented than conventional oils, probably because US induces cell wall rupture, thereby promoting the diffusion of minor compounds such as chlorophylls and other pigments. The US treatments resulted in oils with significantly higher total chlorophyll and carotenoid contents (219 ± 25 and 49 ± 3 (mg/kg), respectively) compared to those of EVOO obtained from untreated olive paste (164 ± 17 and 33 ± 6 (mg/kg), respectively).

Recently, US pre-treatments (35 kHz) of different duration (0, 4, 8, 10 min) of depitted olive paste prior to malaxation were studied together with water supplementation [37]. US treatment did not adversely affect the quality characteristics and oxidative stability of the olive oil and when applied with water resulted in a significant increase in yield for the studied Tunisian and Turkish olive cultivars.

According to Servili et al. [50], the pressure level generated by US on cells has a strong impact on the olive oil extraction process. In a study carried out in olive paste from different olive cultivars (Arbequina, Peranzana, Nocellara del Belice and Coratina) and five comparative tests maintaining the US frequency at 20 kHz, they found a higher extractability when applying a pressure of 3.5 bar compared to the control or the 1.7 bar treatment. No differences were observed regarding olive oil quality (free acidity, peroxide values, K_232_, K_270_, and ΔK) and volatile compounds, whereas the phenolic content increased at 3.5 bar.

To date, most of the studies based on the application US in the olive oil industry have focused on olive fruits or olive pastes. However, only two studies have evaluated the effect of the direct application of this technology on the chemical composition and thermal properties of EVOO. In this sense, US of 40 kHz was applied for 0, 15, 30, and 60 min in virgin olive oil (VOO) of the Arbequina and Picual varieties [42]. The longer the treatment, the higher increase of the oil temperature, but there were no significant effects on the quality parameters (free acidity, K_232_, and K_270_), which led to the conclusion that US does not degrade the oils. Likewise, the US treatment did not alter the lipid profile and the composition of phenols, tocopherols, and pigments (carotenoids and chlorophylls). Regarding the volatile compounds, a slight decrease was observed after 60 min of sonication, which could be explained by the increase in temperature during the treatment. In another study carried out by Femenia et al., the US energy was applied to prevent the total or partial crystallization of EVOO during storage at low temperature, allowing retention of the physical–chemical and sensory properties of the product [62]. 

#### High-Power Ultrasound

In 2007, the effect of HPU on oil yield and quality parameters was evaluated for the first time [40]. Extractability was improved when direct sonication was applied to high moisture olives (> 50%) or indirect sonication to low moisture olives (< 50%). The treatment did not affect the quality parameters (free acidity, peroxide value, K_270_, and K_232_) of EVOO produced from sonicated pastes, whereas the content of tocopherols, chlorophylls, and carotenoids increased. 

Bejaoui et al. [41] tested HPU treatments at three different frequencies (20, 40, and 80 kHz), and EVOOs were extracted after two treatments: HPU application and centrifugation, with or without malaxation. The results demonstrated that HPU treatments had no apparent effect on the fatty acid composition and phenolic content of the EVOO. 

In another study, Arbequina and Frantoio olive pastes were treated directly (110 W/cm^2^ and 19 kHz) or indirectly (150 W/cm^2^ and 20 kHz) for 2, 4, 6, 8, and 10 min by HPU [34]. After treatment, samples were malaxed for 30, 35, 40, and 45 min. HPU was found to increase the olive paste temperature from 20 to 25.5 °C and allowed the optimum temperature of 29 ± 1 °C to be achieved after a shorter malaxation. No significant differences in EVOO yield were found between malaxation times of 35 and 45 min, indicating that HPU could be applied to shorten the process by 10 min. HPU significantly improved EVOO yield by 1% for both varieties, with no significant differences observed in any quality parameters, except the peroxide value, which was slightly higher. Total tocopherol and pigments increased significantly with longer HPU treatments, which generated a darker oil with increased yellow and green color components. The total polyphenols and oxidative stability index decreased after 8 min of HPU treatment. 

The impact of HPU technologies together with the ripening stage and malaxation time on oil yield was also evaluated [35]. No effects were observed in the legal and quality characteristics of VOO, and the commercial category was maintained without significant changes in the product, except for a slight increase in waxes and total sterols in oil produced from fruits with the highest maturity index. The HPU system had a positive impact on VOO production from olive fruits at a ripening early stage, when it was able to exert a highly disruptive effect on cells that were still very physiologically active and therefore induced an abundant release of intracellular content. As a result, a higher extraction yield (22.7%) and phenol content (10.1%) were observed in HPU-VOO compared to the control oil extracted with a traditional process. 

The HPU effect was also assessed with oxygen control during malaxation on a laboratory scale with the aim of improving oil extraction. Low headspace oxygen has been reported to reduce the oil yield due to a lower activity of lipases responsible for breaking the vacuole [63]. With the objective of counteracting this effect, HPU was applied with four different headspace oxygen concentrations (2, 5, 10, and 21%) [36]. The oils produced with oxygen concentrations of 2% and 5% had a lower oxidative index and better sensory attributes, including a more bitter taste, in comparison with oils obtained using more headspace oxygen, in which these parameters did not differ from the untreated control [36].

High frequency US standing waves (megasonics, MS) in the olive oil extraction process were also investigated, evaluating the possible effects of water (0, 15, and 30%), MS power (0, 50, and 100%) and malaxation time (10, 30 and 50 min) [39]. The treatment did not compromise the quality of the EVOO, even at the highest potency. In general, a higher extraction performance was observed with the longer treatments and lower MS power levels. The study showed that long MS treatment of malaxed paste (up to 15 min; 220 kJ/kg) increased the oil extraction capacity by up to 3.2%. The combination of low frequency (40 kHz) sonication to promote cell wall disruption pre-malaxation, followed by post-malaxation MS treatment (585 kHz), improved oil extraction by up to 2.4%. The best results, however, were obtained when the pulp was treated by MS (585 kHz, 10 min, 146 kJ/kg) before malaxation and without water supplementation, which provided an increase in oil extraction capacity of up to 3.8% compared to the non-sonicated control.

### 2.4. Microwave Heating 

**Figure 4:**
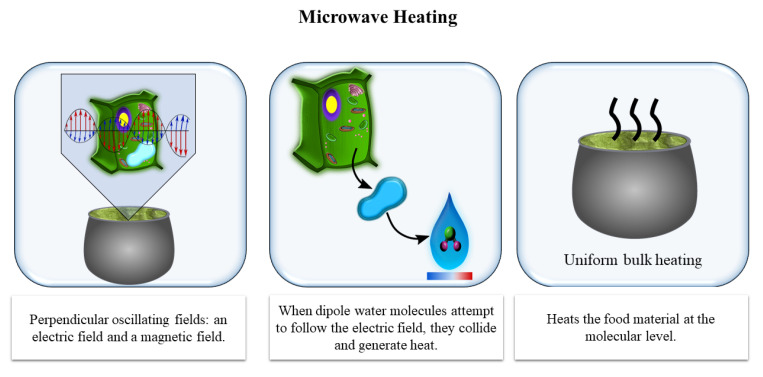
Effect of microwave heating (MW) at the cellular level.

MW heating is based on the high frequency oscillation (several million times per second) between positive and negative electric fields. When the dipole water molecules attempt to follow the electric field, they collide and generate heat, which is rapidly conducted to the surrounding food components (Figure 4). MW energy has long been used for baking, cooking, tempering/thawing, reheating, drying, pasteurization, and sterilization [64]. Its application in food processing can reduce waste, increase throughputs, and improve safety in operations such as thawing frozen meat and fish blocks, precooking food for fast food chains, and pasteurizing pre-packaged foods [65]. The application of MW in the olive oil industry has been little studied to date. 

In a pilot-scale study, Clodoveo et al. applied MW treatment to olive paste to determine if it could improve EVOO extraction yields [33]. The MW process significantly reduced malaxation time, and when applied to olive paste without malaxation the yield was higher than in the control. EVOO quality parameters (acidity, peroxide value, and K_232_ and K_270_) were not affected by the MW treatments. As in the US treatments, EVOO produced from MW-treated olive paste was more pigmented than conventional oils as a result of cell disruption. Values of total chlorophyll and total carotenoids (219 ± 23 and 81 ± 6 mg/kg, respectively) were higher compared to the oils from untreated paste (164 ± 17 and 33 ± 6 mg/kg, respectively). The extraction yield was 16.7% for the conventional process and 17.1% for the MW treatment. When the EVOOs were extracted without malaxation, the MW treatment produced a yield of 5.4%, which was significantly higher than the 1.0% yield of the untreated sample. By inducing cell rupture, the MW application released the oil trapped in the uncrushed olive tissue and thus effectively enhanced oil extraction. 

Leone et al. [43] developed a 4-magnetron microwave tube (2.45 GHz, 24 kW) prototype that allows the rapid and continuous thermal conditioning of 3000 kg of olive paste per hour. The model provided uninterrupted flow and better thermal uniformity than the conventional malaxer. Using this system, the process of thermal conditioning, which in traditional malaxation takes 40 min, was reduced to a few seconds without compromising the oil extraction capacity due to the MW-induced coalescence. In further studies with this MW-assisted process, oil extracted without malaxation had a lower peroxide value than conventionally produced oils due to the short time needed for the heating; it also contained more volatile compounds and a lower amount of phenolic compounds [44]. 

MV has also been combined with MS technology to improve olive oil recovery, a continuous treatment of the olive paste replacing the malaxation step [46,47]. Applied alone, the MW process resulted in an oil with a similar phenolic composition to the control, but when followed by MS, the total phenolic content increased, while total C5 and C6 aldehydes decreased. These promising results have stimulated further developments in this combined continuous MW and MS conditioning technology to optimize extraction yields and total phenolic content in olive oil. The same group created a MW-Heat exchange-US apparatus to improve olive oil extractability [48]. Using this equipment, it was possible to reduce malaxation time from 40 to 20 min, with an increase in the yield and no modification of the total phenolic content or the marketable parameters [49]. The LOX activity and the volatiles concentration was not affected either [66].

## 3. Consumer Acceptance of Olive Oil Processed by Emerging Technologies 

Before launching food products processed with emerging technologies, it is necessary to take into account consumer opinion [67,68]. Research has found that innovative processing techniques are most likely to be accepted by the young and educated people, who perceive environmental friendliness as their main advantage. Negative opinions are related to health concerns, higher prices, insufficient information about the technologies, and a general skepticism [69]. A way to address consumer misgivings would be to provide information about the advantages of new techniques on food labels. Among the new technologies, HPP has the most potential in the next 5–10 years, followed by MW and PEF. 

### 3.1. Pulsed Electric Fields

PEF treatments are accepted as safe by consumers, as no dangerous chemical reactions are involved [70], and the treated products are perceived as more natural compared to conventionally processed food. PEF techniques are also positively viewed as energy-saving and environmentally friendly [71]. Although PEF-treated food is generally considered as not dangerous or prone to causing allergies, a degree of uncertainty has been reported among some consumers [72] concerning possible side effects of applying electricity to food [73]. Such negative attitudes could be modified by providing consumers with more information about the technology [74].

Regarding the quality of EVOO, sensory analysis has revealed that the application of a PEF treatment does not generate any bad flavor or taste in Arbequina olive oil [22]. Additionally, there was no impairment of the parameters established to measure the level of EVOO quality (acidity, peroxide value, K_232_, and K_270_). A study by Puértolas et al. similarly failed to find a negative impact of PEF on the sensory characteristics of olive oil [24]. Both the control and the PEF-treated olive oils scored a value of 0 for defects, indicating that the evaluators did not perceive any specific or unpleasant taste associated with the PEF treatment. Although the technique improved the oil extractability without altering the main qualitative and organoleptic characteristics of the product, the authors conclude that comparative studies would be desirable with other emerging techniques, such as the use of enzymes or US.

### 3.2. High Pressure Processing

Consumer attitudes to HPP-treated food is usually welcomed [72] as it avoids the use of preservatives. Other positive attributes associated with HPP in comparison with conventional thermal processing are greater naturalness, improved taste, and higher nutritional value [75]. As no reports on toxicity have been published, public awareness about HPP is low [70,71,72,73]. Potential consumer concerns could be reduced by including information about HPP on food labels [76], including its advantages and benefits. To the best of our knowledge, only one author has studied consumer acceptability of HPP treatment when applied to improve EVOO extraction. The process increased the oxidative stability of olive oils without any negative impact on their flavor, color, and consistency [23].

### 3.3. Ultrasound Technology

US is considered an environmentally friendly technology because it generates no waste and is not toxic to humans [77]. In fact, HPU has been applied in various industrial sectors, including those related to food processing and food safety. Over the past decade, US treatment has become an alternative non-thermal food processing technique with a growing number of potential applications in the food industry and overall neutral to good acceptability of the final product. 

The effect of HPU on the sensory characteristics of olive paste was first analyzed by Jiménez in 2007 [40], who found that the resulting oils were significantly less bitter than the untreated ones, and no volatiles with an unpleasant taste were detected. A sensory panel test described the US-treated oils as more fruity, green and pungent, and less bitter than the control. Similar results were found by Clodoveo (2013), who established that the lower polyphenol concentration improved the taste of Coratina EVOO, rendering it less bitter and pungent without affecting the fruity notes [31]. In contrast, Almeida et al. (2017) [78] concluded that US application to EVOO processing had improved its the key positive sensory attributes (fruity, bitter, and pungent) by significantly increasing the content of phenols (mainly secoiridoids) and volatile compounds (C6 aldehydes, C6 alcohols, C5 alcohols, C5 dimers). In another study, the sensory analysis showed no differences between commercially available and HPU-treated samples of Arbequina and Frantoio olive oil [34]. Bejaoui [41] reported that volatile compounds linked to positive sensorial attributes had levels similar to those of oils produced by conventional malaxation, whereas those related to off-flavors did not develop. Furthermore, in recent studies EVOO extracted with US showed acceptance among consumers, who were prepared to buy it, albeit without paying more [79,80].

### 3.4. Microwave Heating

Although MW is an emerging technology in food processing, it is already familiar to consumers through the widespread domestic use of MW ovens [65]. Nevertheless, MW-processed food still has some negative associations, considered as potentially harmful for health and often associated with radiation [81]. Overall, however, consumer acceptance of MW-treated foods is high, and the application of this technology will continue to grow [82].

## 4. Emerging Food Technologies for Increasing the Sustainability of the Olive Oil Process

The environmental impact of olive oil production is distributed among the seven stages of the manufacturing process: olive production, destemming, washing, crushing, malaxation, decantation, and separation. The olive production stage, which includes the agricultural practices, is the greatest contributor. Although the application of PEF to improve olive oil extraction does not directly alter the environmental impact of the other stages, its enhancement of yield distributes the impact over more liters of oil. Thus, if the extraction yield grows by 5%, the environmental impact is correspondingly reduced by 5%. Moreover, the electricity consumption of the PEF apparatus is minimal compared to other manufacturing procedures [83].

According to a survey carried out among food managers, scientists, and technologists working in food processing companies, HP is the most widely used novel non-thermal food stabilizing technique in the USA, whereas PEF has greatest usage in beverage, oil, and fat processing companies. The main reason companies implement innovative food technologies is to obtain better nutrient and sensory quality in food (71.14%), whereas only 13.4% of the participants stated water and energy savings [21]. In the case of MW technology, a study showed that it was 24% more energy-demanding than conventional malaxation, but it was still viable because it was less time-consuming and could work in continuous mode [45].

Although the issue of sustainability is a trending topic of great concern, there is a lack of research about the ecological impact of these emerging technologies, generally regarded as greener than conventional processes [84]. In the field of olive oil, new studies are required to assess to what extent their application could resolve the problem of OMW generation.

One of the most studied solutions to deal with the generation of OMW is to develop a circular economy, where the residues are reused and incorporated into a new production cycle. On the one hand, this approach reduces the environmental impact of the OMW, and on the other, it gives added value to the residues. Many valorization options have been suggested, such as composting and soil applications, use as cattle feed, methane production, bioactive compound extraction, and bio-char production [85].

Traditionally, solid-liquid extraction has been used to recover bioactive compounds from food byproducts, but this methodology is time-consuming and unsustainable. Alternative emerging technologies provide advantages in that they can shorten the extraction time, work at lower temperatures, reduce the usage of organic solvents, and improve the extraction yield and quality [86].

A study on the extraction of high-value compounds (polyphenols, flavonoids, and proteins) from olive pomace explored whether PEF and HP achieved better results than solid-liquid extraction [87]. Samples pre-treated with either PEF or HP both contained higher concentrations of polyphenols and proteins, which increased with treatment intensity. The phenolic concentration increased by up to 91.6% and 71.8% when PEF and HP were applied, respectively. The conditions that allowed the highest recovery of polyphenols and proteins with the lowest extraction time (10 min) were PEF (3 kV/cm and 45 ms, with an energy input of 10.9 kJ/kg), and HP (200 MPa and 10 min treatment time, with an energy input of 6.41 kJ/kg). Moreover, these conditions also improved the extraction yield of some individual phenolic compounds, being higher in the case of PEF treatment.

Olive pomace also contains cellulose and hemicellulose, making it a potential source of ethanol via fermentation. A model was proposed for the olive industry in which olive pomace is exploited for ethanol production and the solid remnants as a sorbent of heavy metals from wastewaters [85]. The olive mill solid waste (OMSW) was previously treated with MW (140 °C, 250 psi, 10 min) or autoclaved (121 °C, 17.6 psi, 10 min) and additives (2% H_2_SO_4_ or 0.6 M formic acid or distilled water). The MW pre-treatment resulted in a better saccharification efficiency and sugar release than the autoclave, the highest saccharification yield being obtained with MW and formic acid. This pre-treatment also gave the highest ethanol concentrations after the fermentation step. Finally, the ability of the OMSW solid remnants to absorb heavy metals (Cu and Pb) from water was demonstrated.

Another way of using olive pomace as a renewable energy source is through thermochemical conversion, such as torrefaction or pyrolysis. In conditions of low oxygen content and atmospheric pressure and a temperature of 220 to 500 °C, the biomass decomposes to three main products, bio-char, bio-oil, and bio-gas, which may be used for energy production as bio-fuel [88]. An advantage of applying these technologies as a pre-treatment before other conversion processes is a reduction in waste volumes; the higher the temperature, the greater the mass loss [89].

In a recent study, MW technology was introduced to the pyrolysis process [90] and found to greatly enhance the loss of mass. When applying less energy (between 0.88 and 1.94 kJ/g), the mass loss increased with MW power, and the highest yields of bio-oil were achieved with the lowest input of 150 W. With higher energies (from 2.27 to 3.27 kJ/g), the maximum bio-oil was obtained at 450 W. Overall, the 150 W power input generated the greatest mass loss and bio-oil yields of the pyrolysis process. Moreover, the use of MW did not alter the bio-oil composition, and MW at 200 W resulted in a bio-char with a higher capacity for methylene blue dye adsorption, thus outperforming the conventional heating process.

In the treatment of olive mill wastewater (OMWW), oxidation processes are commonly used to eliminate environmentally toxic and harmful compounds. One of the techniques applied for this purpose is US. A study by Al-Bsoul et al. [91] showed that the combination of US with TiO_2_ nanoparticles as a catalyst was more efficient than using US alone. Alternatively, OMWW can serve as a substrate for edible filamentous fungi, which can be used as a protein source. However, the process still needs to be optimized to increase the production of fungal proteins above the 15% yields currently achieved [92].

All these recent studies reflect the interest and concern for finding efficient and economic methods to reduce the generation of OMW and attenuate its environmental impact. However, this is still a novel field that requires far more extensive research to achieve optimal and sustainable solutions.

## 5. Conclusions

The extraction process assisted by PEF, HPP, US, and MW technologies has proved to be very efficient on olive pastes, leading to a significant increase of the oil yield. Regarding to the content of bioactive compounds (phenols, phytosterols, tocopherol, vitamin E, carotenoids, chlorophylls, and volatile compounds), the oil quality parameters (oxidative stability and peroxide value) and sensory attributes, in general, are improved after extraction process assisted by those emerging technologies, but the degree of the amelioration seems to be dependent on the technology and process conditions used.

For consumers, not only is the quality of olive oil important but also its safety and environmental impact. Often this can lead to consumer demand for information concerning the safety and benefits of these emerging technologies, as well as the environmental impact. As these are emerging technologies, studies and surveys indicate that both consumers and food industries are more willing to accept them if their reviews are positive regarding these aspects. Therefore, there is a tangible motive for scientists and researchers both to prove the safety and innocuousness of these technologies as well as to demonstrate the advantages of the environmental impact when compared to the conventional techniques. Through this and proper dissemination of the scientific conclusions to the consumers can we promote trust and embracement of these emerging technologies.

The application of emerging technologies to enhance mechanical olive oil extraction requires further research on both the establishment of the optimal treatment conditions and the effects of external factors, such as the cultivar, maturity index, and temperature. Nevertheless, they are promising alternatives to conventional processes, not only in terms of enhanced oil extraction but also sustainability. Their advantages may be harnessed to improve oil production and the sustainability of the process.

The technologies described in this review have also been applied to OMW. Although the number of studies is limited, these technologies seem to also have positive effects in reducing the quantity of residues and revalorizing them. Nevertheless, future in-depth research should be focused on the benefits of using these technologies for the valorization of OMW.

## Figures and Tables

**Table 1:** Effect of emerging technologies of yield, quality parameters and bioactive compounds from extra virgin olive oil (EVOO).

Technologies	Cultivar	Parameters	Matrix of Application	Effect	Ref
**PEF**	Arroniz	11.25 kJ/kg.	Olive paste after malaxation	Increased extraction yield (13.3%). Increased TPC, phytosterol, and tocopherol contents.	[24]
	Carolea, Coratina, and Ottobratica	17 kJ/kg.		Increased extraction yield (2.3–6%).Increased TPC (3.2–14.3%).	[13]
	Arbequina	1.47–5.22 kJ/kg.	Olive paste before malaxation	Increased extraction yield (14%) without or with malaxation at 15 °C. Reduced TPC and unaltered vitamin E.	[22]
	Unspecified	7.83 kJ/kg.		Increased extraction yield (7.5%). Increased oleacein and oleocanthal concentration at low temperature.	[25]
	Coratina	16 kV, 100 µs pulse duration		Increased oil extractability (3.71%). Increased oil yield (0.38%).	[26]
	Tsounati, Amfissis, Manaki	1.6–70.0 kJ/kg.	Fruit	Increased extraction yield (18%). Increased TPC. Improved oxidative stability.	[23]
	Unspecified	0.7 kV/cm (30 pulses) 1.3 kV/cm (100 pulses)		Increased extraction yield (7.4%) at 1.3 kV/cm.	[27]
**HPP**	Tsounati, Amfissis, Manaki	200 and 600 MPa for 1 and 5 min.	Fruit	Increased extraction yield (16%). Increased TPC. Improved oxidative stability.	[23]
	Frantoio	608 MPa for 6 min.	Filtered and unfiltered oil	Less fusty and rancid sensory attributes when the oil was unfiltered; no differences for the filtered oil.	[28]
**US**	Coratina	0.4 and 2 MHz, 280 W, 2.5 and 5 min	Olive paste after malaxation	Increased yield in all cases (10%).	[29]
	Unspecified	150 W, 30 kHz, 120–300 s	Olive paste before malaxation.	Improved sensory evaluation. Increased tocopherol, carotenoids, and phenolic compounds. Reduced polyphenol oxidase activity.	[30]
	Coratina and Peranzana	150 W 35 kHz 2–10 min		Reduced malaxation time. Increased carotenoid, chlorophyll, and tocopherol content. Reduced TPC.	[31]
	Memecik and Chemlali	150 W, 35 kHz, 4–10 min		Increased secoiridoids concentration.	[32]
	Ogliarola Barese	150 W, 35 kHz, 10 min		Increased extraction yield (17%).	[33]
**US**	Arbequina and Frantoio	Directly: 110 W/cm^2^ 19 KHz Indirectly: 150 W/cm^2^ 20 kHz 2–10 min		Increased extraction yield (1%). Increased tocopherols, pigments, and peroxide value. Decreased TPC and oxidative stability index (in treatments longer than 8 min). The treated oil was darker.	[34]
	Ogliarola garganica	2.8 kW 20 kHz, continuous 2 tons/h		Increased extraction yield, especially with less ripe olives (22%); increased TPC.	[35]
	Arbequina	150 W, 20 kHz, 6 min		Increased extraction yield (10%). Increased tocopherols, carotenoids, and chlorophylls content.	[36]
	Chemlali and Memecik	150 W, 35 kHz 4–10 min		Increased yield with increasing US treatment time. No changes in oil composition or oil stability.	[37]
	Coratina	4 kW		Increased oil extractability (3.57%). Increased oil yield (0.54%).	[26]
**US**	Coratina	150 W 35 Hz 10 min	Fruit and olive paste before malaxation	Increased extraction yield (6.2%). Increased chlorophylls, carotenoids, tocopherols, and phenolic content.	[38]
		36-146 kJ/kg, 20–600 kHz	Olive paste before, during, and after malaxation	Increased extraction yield, especially when US was applied before and after malaxation (4%).	[39]
	Picual	105 W/cm^2^ 24 Hz and 150 W/cm^2^ 25 Hz 0–30 min	Olive paste during malaxation, directly to the paste and to the water bath, respectively	Increased tocopherols, carotenoids, and chlorophylls content. Decreased TPC. Improved attributes in the sensory analyses.	[40]
		900 W, 20–80 kHz in continuous mode: 200 kg/h	Olive paste before malaxation or before centrifugation when no malaxation was performed	More balanced sensorial profile. Increased secoiridoids levels and (E)-2-hexenal.	[41]
	Arbequina and Picual	150 W, 40 kHz, 15–60 min	EVOO	Slight decrease in individual volatile compounds.	[42]
**MW**	Coratina	24 kW at 2.45 GHz continuous: 3tones/h.	Olive paste before malaxation or before centrifugation when no malaxation was performed.	Increased coalescence. Increased volatiles compounds. Decreased TPC and peroxide value.	[43,44,45]
	Ogliarola garganica			Increased extraction yield (1.8%). Increased TPC when megasound was applied.	[46,47]
	Ogliarola Barese	800 W 180 s 2.45 GHz	Olive paste before malaxation	Increased extraction yield.	[33]
**Combination**	Coratina	MW 3.3–4.0 kW 2.45 GHz 395 kg/h + US 2.08 kW 400–600 Hz 395 kg/h	Olive paste before malaxation or before centrifugation when no malaxation was performed	The combination of MW and US obviated the need for malaxation and increased the yield (2.2%). Increased TPC when megasound was applied.	[46,47]
		MW 5.34 kW, 2.45 GHz 1200 kg/h + US 3.3 kW, 20 kHz, 1200 kg/h + heat exchange		The combination of US, MW, and a spiral heat exchange device achieved a higher yield than conventional extraction. No changes observed in the oil.	[48,49]
**Combination**	Arbequina, Peranzana, Nocellara, Coratina	HPP 1.7–3.5 Bar + US 2.6–3.5 kW, 20 Hz, 2300 kg/h	Olive paste before malaxation	Increased extraction yield (6%). Increased TPC.	[50]

TPC: total phenols content; US: ultrasound; MW: microwaves.
